# The burden attributable to primary headache disorders in children and adolescents in Ethiopia: estimates from a national schools-based study

**DOI:** 10.1186/s10194-024-01743-0

**Published:** 2024-04-02

**Authors:** Yared Zenebe Zewde, Mehila Zebenigus, Hanna Demissie, Redda Tekle-Haimanot, Derya Uluduz, Tayyar Şaşmaz, Fatma Bozdag, Timothy J Steiner

**Affiliations:** 1 Department of Neurology, School of Medicine, College of Health Sciences, Addis Ababa University, Addis Ababa, Ethiopia; 2 Department of Internal Medicine, School of Medicine, College of Health Science, Addis Ababa University, Addis Ababa, Ethiopia; 3 Neurology Department, Cerrahpaşa School of Medicine, Istanbul University, Istanbul, Turkey; 4 Public Health Department, School of Medicine, Mersin University, Mersin, Turkey; 5 Siirt Kurtalan District Health Directorate, Kurtulan, Turkey; 6 NorHEAD, Department of Neuromedicine and Movement Science, Norwegian University of Science and Technology, Trondheim, Norway; 7 Department of Neurology, University of Copenhagen, Copenhagen, Denmark; 8 Division of Brain Sciences, Imperial College London, London, UK

**Keywords:** Child and adolescent headache, Migraine, Tension-type headache, Medication-overuse headache, Undifferentiated headache, Epidemiology, Burden of disease, Schools-based study, Ethiopia, Sub-Saharan Africa, Global campaign against headache

## Abstract

**Background:**

We previously reported high prevalences of headache disorders among children (6–11 years) and adolescents (12–17 years) in Ethiopia. Here we provide data on headache-attributed burden collected contemporaneously from the same study participants. Part of the global schools-based programme within the Global Campaign against Headache, the study is the first to present such data from sub-Saharan Africa.

**Methods:**

A cross-sectional survey following the generic protocol for the global study was conducted in six schools (urban and rural), in Addis Ababa city and three regions of Ethiopia. The child or adolescent versions of the Headache-Attributed Restriction, Disability, Social Handicap and Impaired Participation (HARDSHIP) structured questionnaires were self-completed under supervision by pupils in class. Headache diagnostic questions were based on ICHD-3 beta but for the inclusion of undifferentiated headache (UdH).

**Results:**

Of 2,349 eligible participants, 2,344 completed the questionnaires (1,011 children [43.1%], 1,333 adolescents [56.9%]; 1,157 males [49.4%], 1,187 females [50.6%]; participating proportion 99.8%). Gender- and age-adjusted 1-year prevalence of headache, reported previously, was 72.8% (migraine: 38.6%; tension-type headache [TTH]: 19.9%; UdH: 12.3%; headache on ≥ 15 days/month (H15+): 1.2%). Mean headache frequency was 2.6 days/4 weeks but, with mean duration of 2.7 h, mean proportion of time with headache was only 1.0% (migraine: 1.4%; TTH: 0.7%; H15+: 9.1%). Mean intensity was 1.8 on a scale of 1–3. Symptomatic medication was consumed on about one third of headache days across headache types. Lost school time reportedly averaged 0.7 days over the preceding 4 weeks, representing 3.5% of school time, but was 2.4 days/4 weeks (12.0%) in the important small minority with H15+. However, actual absences with headache the day before indicated averages overall of 9.7% of school time lost, and 13.3% among those with migraine. Emotional impact and quality-of-life scores reflected other measures of burden, with clear adverse impact gradients (H15 + > migraine > TTH > UdH).

**Conclusions:**

The high prevalence of headache among children and adolescents in Ethiopia, who represent half its population, is associated with substantial burden. Lost school time is probably the most important consequence. Estimates suggest a quite deleterious effect, likely to be reflected in both individual prospects and the prosperity of society.

## Introduction

Studies in Turkey [1], Austria [2], Lithuania [3], Iran [4], Mongolia [5] and Zambia [6], all employing similar schools-based methodology and survey instruments [7], have reported highly prevalent primary headache disorders among children (aged 6–11 years) and adolescents (aged 12–17 years). Migraine, tension-type headache (TTH) and undifferentiated headache (UdH) – defined as short-duration (< 1 h) mild headache, meeting criteria for neither migraine nor TTH [1] – were all common. We followed the same protocol in Ethiopia, finding an age- and gender-adjusted 1-year prevalence of all headache of 72.8%, but reporting diagnostic uncertainties, particularly between probable migraine and UdH [8].

Three factors were likely to have contributed to these uncertainties. First, the differential diagnosis between UdH and probable migraine often hinges on a 1-point difference in a subjective 3-point intensity scale [1, 9] or on accurate recall and reporting of duration within a quite narrow range (in these age groups, migraine may last no more than 2 h [10], and probable migraine less). Second, given that UdH is believed to represent expressions of migraine or TTH by the immature brain [1], clear distinction is not always to be expected. Third, questionnaire-based enquiry of young age groups, even when mediated by their teachers, has a level of inbuilt and unavoidable unreliability [7]. These same uncertainties were encountered in Zambia [6], also in sub-Saharan Africa (SSA), although not in the studies elsewhere [1–5].

Regardless of diagnosis, headache affecting almost three quarters of children and adolescents raises concerns about impacts on health, education and future prospects in a country where almost half of the population are aged under 18 years [11]. However, as a measure, prevalence alone is not informative about these impacts. Measures of disease-attributed burden are needed also [9]. This study provides these data, with the purposes of informing local health and educational policies, and of contributing to knowledge of the global burden of headache [12, 13]. As were the studies elsewhere [1–6], it was conducted as a project within the Global Campaign against Headache, under the direction of *Lifting The Burden* (LTB) [14–17], as part of its global programme of similar schools-based studies [7, 17].

## Methods

Details of the methodology have been published previously [7, 8]. Data on headache-attributed burden were collected contemporaneously with those on prevalence, from the same participants, in a cross-sectional survey among pupils in schools selected to be nationally representative [8].

### Ethics and approval

The study protocol was approved by the Institutional Review Board of the College of Health Sciences of Addis Ababa University. In addition, authorization letters were obtained from each Regional Education Bureau.

Principals and teachers at each selected school gave their agreement to participation. Prior consent was obtained from or on behalf of each participating pupil.

Data were collected anonymously, and held in compliance with data protection legislation.

### Sampling and recruitment

We conducted the survey during one academic term in 2018 in six randomly selected schools located in four regions of Ethiopia, the latter purposively selected to capture the socioeconomic, cultural and geographic diversities of the country [8].

In each school, we included all pupils from each class aged between 6 and 11 years and/or 12–17 years who were present on the day of the survey and by or for whom consent had been given. Following published recommendations [7, 9], we aimed to recruit 200 evaluable participants of each age in the range 6–17 years to achieve *N* = 2,400.

### Survey instruments and data collection

We used the child and adolescent versions of LTB’s Headache-Attributed Restriction, Disability, Social Handicap and Impaired Participation (HARDSHIP) structured questionnaire [7], translated into Oromiffa (Afan Oromo) and Amharic languages, the two commonly used languages in Ethiopia. These questionnaires were administered in class and completed by pupils under the supervision of an investigator and teacher, with younger children assisted when necessary. Teachers received additional questionnaires enquiring into relevant school variables. All data were collected during a single academic year, avoiding examination periods. Further details have been published earlier [7, 8].

Diagnoses were made by algorithm applied to survey questions [18] and, apart from UdH [1], were based on the criteria of the *International Classification of Headache Disorders* (ICHD), version 3 [10].

Burden enquiry included symptom burden (frequency of headache, and its usual duration and intensity during episodes), symptomatic medication intake (frequency), lost time from schooling and other activities as well as lost parental work time (using adaptations of the Headache-Attributed Lost Time (HALT) indices [19]), and selected (headache-relevant) questions from KINDL® [20] addressing concentration, emotional impact and quality of life (QoL) [7]).

### Data entry and analysis

All completed questionnaires were moved to Addis Ababa University, where two independent data entries into SPSS statistical package (version 25) were performed, and discrepancies resolved by comparison with source data.

Data analysis was conducted at the University of Mersin, Turkey.

Reported headache frequency [F] was expressed in terms of days in the preceding 4 weeks, and medication intake in days in the preceding week and 4 weeks. Headache intensity, reported categorically as “mild”, “moderate” or “severe”, was expressed on a scale of 1–3. Duration of episodes [D], reported in hours as < 1, 1–2, 2–4 or > 4, was expressed according to the mid-points (0.5, 1.5, 3 and 8 h respectively, assuming a range of 4–12 h for the last). Proportion of time in ictal state (pTIS) was calculated according to the formula (pTIS = F/28*D/24). Lost school time because of headache in the preceding 4 weeks was expressed in days/20 days, assuming a 5-day school week, counting absence from school as a whole day and leaving early as a half-day. Days of limited activity (defined as “could not do things I wanted to because of my headaches”) were counted separately. We recorded the proportion of parents (one or other) who had reportedly lost time from their own work during the preceding 4 weeks because of their son’s or daughter’s headache, regardless of how much (participants were not expected to be able to report this reliably).

Participants reporting headache on the day prior to the survey (“headache yesterday” [HY]) were counted, and proportions calculated of the total sample and of those with each headache type. Intensity of HY was expressed on the same 3-point scale as above. Participants reporting a lost school day because of HY were also recorded as proportions, which were then compared with predicted estimates, calculated as the product of number affected by each headache type and mean reported headache days (divided by 20) lost per pupil over the preceding 4 weeks.

Responses to questions addressing concentration, emotional impact and QoL were all on a 4-point Likert scale (“never”, “sometimes”, “often”, “always”) [7], also referring to the preceding 4 weeks. We scored these 0–3, and summed them to generate an emotional impact score (including concentration; potential range 0–18, high being adverse) and a QoL score (0–36, low being adverse).

We expressed proportions as %, calculating 95% confidence intervals (CIs), and used chi-squared tests for comparisons. We treated all other variables as continuous, and used descriptive statistics (means and standard deviations [SDs]). For comparisons of continuous data (which were not distributed normally), we used Kruskal-Wallis test with *post hoc* Dunn test.

In all analyses we considered *p* < 0.05 to be significant.

## Results

There were 2,349 eligible pupils present on the survey days (males 1,162; females 1,187), all of whom agreed to participate, but five males were excluded because of incomplete responses. The number analysed was therefore 2,344 (males 1,157 [49.4%]; females 1,187 [50.6%]). These included 1,011 children (43.1%) and 1,333 adolescents (56.9%), with an overall mean age of 12.0 ± 2.2 years (median 12) [8]. The participating proportion was 99.8%.

As previously reported, the gender- and age-adjusted 1-year prevalence of any headache was 72.8%, higher among adolescents (77.6%) than children (68.4; *p* < 0.001) [8]. Age-adjusted 1-year prevalence of migraine was 38.6% (of which 22.5% was probable), of TTH 19.9%, of UdH 12.3%, of all headache on ≥ 15 days/month (H15+) 1.2%, and of probable medication-overuse headache (pMOH) 0.2% [8].

### Symptom burden

Table 1 shows reported headache frequency, duration and intensity for headache overall and by headache type, along with estimates for each of pTIS. None of these data were normally distributed.

**Table 1:** Symptom burden overall and by headache type (*N* = 2,344)

**Headache type**	**n**	**Mean frequency** (days/4 weeks)	**Mean duration** (hours)	**Estimated proportion of time in ictal state** (%)	**Mean intensity** (scale 1–3)^a^
All headache	1,726	2.6 ± 3.2	2.7 ± 2.8	1.0	1.8 ± 0.7
Migraine	900	2.8 ± 2.9	3.6 ± 3.0	1.4	2.2 ± 0.6
Tension-type headache	477	2.0 ± 2.3	2.4 ± 2.3	0.7	1.7 ± 0.6
All headache on ≥ 15 days/month^b^	29	16.1 ± 2.6	3.8 ± 3.4	9.1	2.1 ± 0.8
Undifferentiated headache	298	1.8 ± 2.3	0.5 ± < 0.1	0.1	1.0 ± < 0.1

Means are presented ± SDs

^a^Equating 1-3 to the reported categories “mild”, “moderate” and “severe”, and treating as continuous data.

^b^There were too few cases of probable medication-overuse headache to analyse separately

Frequency of headache overall was 2.6 days/4 weeks. H15 + was, of course, far more frequent than the episodic headaches. Among the latter, TTH (*p* < 0.01) and UdH (*p* < 0.001) were less frequent than migraine. Headache episodes were relatively short-lasting, with migraine of longer duration (3.6 h) than TTH (2.4 h; *p* < 0.001), while the overall mean of 2.7 h was brought down by UdH (0.5 h: Table 1). Mean pTIS was, accordingly, low overall (1.0%), and very low for UdH (0.1%), but reached 9.1% for H15+ (Table 1) and 17.2% for pMOH (not shown).

Mean headache intensity was 1.8 overall (mild-to-moderate), greater for migraine (2.2) than TTH (1.7; *p* < 0.001) (Table 1). UdH, mild by definition, was rated less intense (1.0) than both migraine and TTH (*p* < 0.001).

### Medication use

Symptomatic medication use is shown in Table 2. In all cases except for H15+, intake recalled over the preceding 4 weeks (with overall mean of 1.0 days) was less than expected from intake reported in the preceding one week. Based on the 4-week data, medication was taken more often for migraine than for TTH (*p* < 0.01) or UdH (*p* < 0.001) and, as expected, much more frequently for H15 + than for all other headache types (*p* < 0.001) (Table 2).

**Table 2:** Symptomatic medication use (frequency) overall and by headache type (*N* = 2,344)

**Headache type**	**n**	**Preceding week**	**Preceding 4 weeks**	**Headache frequency in preceding 4 weeks**
		days (mean ± SD)		
All headache	1,726	0.6 ± 1.1	1.0 ± 2.0	2.6 ± 3.2
Migraine	900	0.7 ± 1.1	1.1 ± 1.9	2.8 ± 2.9
Tension-type headache	477	0.5 ± 0.9	0.7 ± 1.5	2.0 ± 2.3
All headache on ≥ 15 days/month	29	1.6 ± 2.0	6.2 ± 5.7	16.1 ± 2.6
Undifferentiated headache	298	0.4 ± 0.9	0.6 ± 1.3	1.8 ± 2.3

Table 2 repeats the data for headache frequency for comparison. Across headache types, medication was used on only about one third of headache days.

### Lost useful time

These data, together with headache frequencies from Table 1 for comparison, are shown in Table 3. Lost school time per affected pupil because of any headache averaged 0.7 days/4 weeks (i.e., 0.7 days in 20 [3.5%]). Lost school time was greater for migraine than for TTH (*p* < 0.01) or UdH (*p* < 0.001), and greater for H15+ (costing those affected 12% [2.4/20] of their school days) than for all other headache types (*p* ≤ 0.001).

**Table 3:** Lost useful time because of headache in the preceding 4 weeks, overall and by headache type (*N* = 2,344)

**Headache type**	**n**	**Headache frequency**	**Lost school time**	**Limited activity**^a^	**Parent missed work**^a^
		days/4 weeks/affected pupil (mean ± SD)			n (%)
All headache^b^	1,726	2.6 ± 3.2	0.7 ± 1.5	1.0 ± 1.8	245 (14.2)
Migraine^b^	900	2.8 ± 2.9	0.9 ± 1.7	1.2 ± 1.8	154 (17.1)
Tension-type headache	477	2.0 ± 2.3	0.5 ± 1.1	0.7 ± 1.3	47 (9.9)
All headache on ≥ 15 days/month	29	16.1 ± 2.6	2.4 ± 2.7	5.0 ± 5.2	10 (34.5)
Undifferentiated headache	298	1.8 ± 2.3	0.4 ± 1.3	0.6 ± 1.1	31 (10.4)

^a^See text for explanation

^b^One pupil did not provide a response regarding lost school time or limited activity

For all except H15+ (affecting > 1 day in every six [5.0/28]), the proportions of days reported with limited activity were similar to those lost from school, allowing for a denominator for the former of 28 rather than 20.

One in seven parents (14.2%) missed work at least once in 4 weeks because of their son’s or daughter’s headache (Table 3), a higher proportion for those with migraine than for those with TTH or UdH (*p* < 0.001). The proportion rose to one in three (34.5%) for those with H15+ (*p* < 0.05 *versus* migraine; *p* < 0.001 *versus* TTH and UdH). As noted, the data collected did not specify amount of time lost.

### Headache yesterday

A total of 630 participants reported HY: one quarter (26.9%) of the total sample and one third (36.5%) of those with any headache (Table 4). This was gender-related: 30.8% of males and 41.7% of females with any headache reported HY (*p* < 0.001; not shown). It was also diagnosis-related, the proportion being highest (as expected) for H15 + and trending downwards between migraine, TTH and UdH (Table 4). Those with migraine or H15 + reported higher-intensity HY than those with TTH or UdH (*p* ≤ 0.001). Participants with migraine and reporting HY were more likely to have lost school time yesterday than those with TTH or UdH and reporting HY (*p* < 0.01).

**Table 4:** Headache yesterday (HY) and lost school time yesterday, overall and by headache type (*N* = 2,344)

**Headache type**	n	**Headache yesterday**			
		**Proportion reporting HY** n (%)	**Intensity of HY** scale 1–3^a^ (mean ± SD)	**Missed school day yesterday** **n (% of those reporting headache type)**	
				**Actual**	**Predicted**
All headache	1,726	630 (36.5)	1.9 ± 0.7	167 (9.7)	60 (3.5)
Migraine	900	366 (40.7)	2.1 ± 0.7	120 (13.3)	41 (4.5)
Tension-type headache	477	157 (32.9)	1.7 ± 0.7	30 (6.3)	12 (2.5)
All headache on ≥ 15 days/month	29	19 (65.5)	2.2 ± 0.6	3 (10.3)	3.5 (12.0)
Undifferentiated headache	298	86 (28.9)	1.5 ± 0.5	13 (4.3)	6 (2.0)

^a^Equating 1-3 to the reported categories “mild”, “moderate” and “severe”, and treating as continuous data

Table 4 compares actual lost school yesterday with predictions based on the 4-week (recalled) data of Table 3 (column 4). Thus, for example, the prediction for all headache was (0.7/20)*1,726 = 60. For all episodic headaches, actual values were always higher (2-3-fold) than predicted values.

### Emotional impact and quality of life (QoL)

Emotional impact scores, with a mean value of 6.2 for all headache, were not normally distributed. Judged nonetheless by means, migraine and H15+, while not significantly different from each other, had greater adverse emotional impact than TTH or UdH (*p* ≤ 0.001) (Table 5).

**Table 5:** Emotional impact and quality of life, overall and by headache type (*N* = 2,344)

**Headache type**	**n**	**Emotional impact score**^**a**^ scale 0–18^a^ (mean ± SD)	**Quality-of-life score**^**a**^ scale 0–36^a^ (mean ± SD)
No headache	618	-	21.6 ± 4.8
All headache	1,726	6.2 ± 3.1	20.2 ± 5.0
Migraine	900	6.8 ± 3.2	18.9 ± 4.8
Tension-type headache	477	5.5 ± 2.8	20.2 ± 4.9
All headache on ≥ 15 days/month	29	8.0 ± 3.3	16.4 ± 4.9
Undifferentiated headache	298	5.0 ± 2.7	21.4 ± 5.0

^a^See text for explanation

QoL scores were also not normally distributed. Again judged by means, all headache types except UdH adversely impacted QoL (*p* < 0.001 *versus* no headache) (Table 5). H15 + had greatest impact, significantly *versus* TTH and UdH (*p* ≤ 0.001) but not *versus* migraine, while migraine had greater impact than TTH (*p* = 0.002) and UdH (*p* < 0.001) (Table 5).

## Discussion

Our previous paper presented prevalence data, finding that headache was very common among children and adolescents in Ethiopia, affecting almost three quarters of these age groups [8]. Migraine was the most often reported type (over one third), followed by TTH (one fifth); UdH, while common at 12.7%, was less so than these specific headache disorders. H15 + was reported by 1.2%, very little of this being pMOH (0.2%) [8]. Here we report the associated burdens.

In summary, mean headache frequency was 2.6 days/4 weeks but, with a mean duration of only 2.7 h, mean pTIS was only 1.0% (1.4% for migraine, 0.7% for TTH and 9.1% for all H15+). Mean intensity was 1.8 on a scale of 1–3 (mild-to-moderate). Symptomatic medication was consumed on about one third of headache days across all headache types. Lost school time reportedly averaged 0.7 days over the preceding 4 weeks, representing 3.5% of school time assuming a 5-day school week, but was 2.4 days/4 weeks (12.0%) in the small but important minority with H15+. Emotional impact and QoL scores reflected these other measures of burden, with clear gradients from H15+ (greatest impact) through migraine and TTH to UdH (least impact).

In our earlier paper, we questioned the diagnoses made of migraine, believing the estimated prevalence of 38.6% to be implausible [8]. More than half was probable migraine, and we commented in that paper, as we do here in our Introduction, on the diagnostic uncertainties particularly between probable migraine and UdH. It is therefore noteworthy that, on all measures of burden, migraine (as diagnosed, and including probable migraine) differentiated clearly from both TTH and UdH. This, perhaps, lends some credence to these diagnoses.

Nevertheless, uncertainty remains. This does not, however, invalidate our burden estimates, even if these may, in part, be questionably attributed to headache type. They remain highly pertinent to public health and to health and educational policies in Ethiopia.

Medication days were fewer than headache days (about one third). This may not be surprising, since durations were short (only 3.6 h even for migraine). Ethiopia is a low-income and very highly ruralised country [21], so access to medications is limited for many. Among those with H15+, medication days were only 6.2 in the preceding 4 weeks, well below the threshold likely to lead to MOH (we recorded only 4 cases [0.2%] of pMOH [8]). Among adults in Ethiopia, only 0.7% report pMOH [22].

Lost school time is probably the most important aspect of headache-attributed burden in these age groups. While estimates based on recall during the preceding 4 weeks were not unduly alarming (averaging 3.5% of school time lost), these values were substantially lower (one third to one half) than those derived from actual absences the day before with HY. The latter might be considered more reliable, since, presumably, they were based on objective observation. They tell a quite different story: an average overall of 9.7% of school time lost, and even higher (13.3%) for those diagnosed with migraine. These estimates predict a quite deleterious effect of headache – and especially migraine – on education, with serious consequences both for individual future prospects and for the prosperity of society. For this finding alone, this study has considerable importance.

Parental work was also a casualty, with one in seven of one or other parent (14.2%) reportedly missing work as a consequence of their son’s or daughter’s headache at least once in the preceding 4 weeks. In a low-income economy, these losses are likely to be troublesome. As might be expected from other measures of burden, parental work losses were strongly influenced by headache type, being three-fold more for H15+, and almost two-fold more for migraine, than for TTH or UdH.

These various burdens were reflected in both emotional impact and QoL scores. The six questions contributing to the former score related principally to concentration, mood, fear of headache and coping with it [7], so the gradient observed – H15 + having greatest impact, followed by migraine and TTH, and UdH with the least – was as expected. QoL scores showed a similar picture, with UdH having no impact. Incidentally but notably, QoL scores were generally low: 60% of the possible score of 36 for those with no headache. While the validation of the full KINDL scale suggests “healthy” scores of about 80% [23], we used only those KINDL questions that seemed relevant to headache [7]. The only comparable published data are from Lithuania, with scores on a similar gradient but averaging 71.2% of maximum possible in those with no headache [24]. It appears that primary headache disorders adversely affect QoL even when other factors cause it to be impaired.

The existence of these gradients across headache types, as already noted, lends some credence to the diagnostic categorisations.

### Strengths and limitations

These were as noted previously [8]. Strengths lay in the adequate sample size [9], the very high participating proportion (99.8%) and the tested and validated methodology [1–7]. The diagnostic uncertainty as a limitation has been acknowledged and discussed, but burden estimates remain informative for local health and education policies even if they may in part be diagnostically misattributed.

Schools-based sampling is valid when education is compulsory and uptake is high [9]. In Ethiopia, primary education is, officially, from 7 to 14 years of age, with school intake nonetheless including some aged 6 years. Intake among those aged 7 years exceeded 90% nationally in 2018/19 [25]. Secondary education is, again officially, from 15 to 18 years, but high drop-out rates from grade 2 (age 8 years) onwards, primarily from low-income families [26], greatly reduce transition from primary to secondary education (nationally, 32.0% in 2018/19: 48.5% for grades 9–10 [15–16 years] and only 14.8% for grades 11–12 [17–18 years] [25]). We acknowledge this possibly important limitation also, but no other sampling method (than schools-based) is likely to afford better access to these age groups [9].

## Conclusions

The high prevalence of headache disorders among children and adolescents in Ethiopia is associated with substantial burden. Lost school time is probably the most important aspect of this. Estimates suggest a quite deleterious effect, with serious consequences both for individual future prospects and for the prosperity of society. Health and educational policies should take note.

## Data Availability

No datasets were generated or analysed during the current study.

## Abbreviations

H15+: Headache on ≥ 15 days/month
HALT: Headache-Attributed Lost Time (indices)
HARDSHIP: Headache-Attributed Restriction, Disability, Social Handicap and Impaired Participation (structured questionnaire)
HY: Headache yesterday
ICHD: International Classification of Headache Disorders
LTB: *Lifting The Burden*
MOH: Medication-overuse headache
pMOH: probable MOH
pTIS: proportion of time in ictal state
QoL: Quality of life
SSA: Sub-Saharan Africa
UdH: Undifferentiated headache
